# The relationship between the effect-site concentration of propofol and sedation scale in children: a pharmacodynamic modeling study

**DOI:** 10.1186/s12871-021-01446-y

**Published:** 2021-09-09

**Authors:** Young-Eun Jang, Sang-Hwan Ji, Ji-Hyun Lee, Eun-Hee Kim, Jin-Tae Kim, Hee-Soo Kim

**Affiliations:** Department of Anesthesiology and Pain Medicine, Seoul National University Hospital, College of Medicine, Seoul National University, Seoul, Republic of Korea, #101 Daehak-no, Jongno-gu, 03080 Seoul, Republic of Korea

**Keywords:** Anesthesia, Anesthetics, Pharmacodynamics, Pharmacokinetics, Sedation

## Abstract

**Background:**

Continuous infusion of propofol has been used to achieve sedation in children. However, the relationship between the effect-site concentration (*C*_*e*_) of propofol and sedation scale has not been previously examined. The objective of this study was to investigate the relationship between the *C*_*e*_ of propofol and the University of Michigan Sedation Scale (UMSS) score in children with population pharmacodynamic modeling.

**Methods:**

A total of 30 patients (aged 3 to 6 years) who underwent surgery under general anesthesia with propofol and remifentanil lasting more than 1 h were enrolled in this study. Sedation levels were evaluated using the UMSS score every 20 s by a 1 μg/mL stepwise increase in the *C*_*e*_ of propofol during the induction of anesthesia. The pharmacodynamic relationship between the *C*_*e*_ of propofol and UMSS score was analyzed by logistic regression with nonlinear mixed-effect modeling.

**Results:**

The estimated *C*_*e50*_ (95% confidence interval) of propofol to yield UMSS scores equal to or greater than *n* were 1.84 (1.54–2.14), 2.64 (2.20–3.08), 3.98 (3.66–4.30), and 4.78 (4.53–5.03) μg/mL for *n* = 1, 2, 3, and 4, respectively. The slope steepness for the relationship of the *C*_*e*_ versus sedative response to propofol (95% confidence interval) was 5.76 (4.00–7.52).

**Conclusions:**

We quantified the pharmacodynamic relationship between the *C*_*e*_ of propofol and UMSS score, and this finding may be helpful to predict the sedation score at the target *C*_*e*_ of propofol in children.

**Trial registration:**

http://www.clinicaltrials.gov (No.: NCT03195686, Date of registration: 22/06/2017).

## Background

Procedural sedation induces anxiolysis, unconsciousness, and analgesia for patient’s comfort. Pediatric patients often require sedation for examination, brief procedures, and imaging studies because of anxiety, fear, distress, and agitation owing to parental separation [1]. Propofol is widely used for pediatric procedural sedation as it has a potent dose-dependent hypnotic action [2–6]. Additionally, propofol reduces airway reflex, postoperative nausea and vomiting, and emergence delirium in pediatric patients and is necessary for children susceptible to malignant hyperthermia [7, 8]. The important issues during propofol sedation are avoiding overdosage or underdosage [9, 10], and maintaining adequate spontaneous ventilation and vital signs [5, 11–13].

For longer procedural sedation, continuous infusion of propofol should be used [14, 15]. Several studies have investigated the manual infusion dosage required to achieve sedation in children [7, 14, 15]. However, as the central volume of distribution and clearance of propofol changes during development, manual infusion of propofol need age-specific dosage adjustment [7, 8, 10, 14–16]. Additionally, manual infusion of propofol guided by clinical assessment of the sedation scale is associated with an increased risk of overdosage or underdosage when compared to that with target-controlled infusion (TCI) [8–10]. Hypotension, bradycardia, apnea, airway obstruction, and delayed recovery are associated with overdosage of propofol [11, 17, 18] while insufficient concentrations of propofol can result in awakeness, sympathetic stimulation, and unsatisfactory procedural condition [10, 11].

TCI of propofol has been used in anesthesia for more than 20 years, and its use has spread widely because of the convenience in usage, stable blood concentration estimations, and rapid recovery it offers [10, 11, 19, 20]. The development of pediatric TCI models for propofol, such as those by Kataria et al. [21], Absalom et al. [22], and Choi et al. [16] has led to the widespread usage of TCI of propofol for inducing sedation and general anesthesia in children [8, 10, 12, 16, 23–25].

Recently, studies have shown that TCI of propofol has a more stable bispectral index (BIS) within the target range with less dose adjustment than that of manual infusion during general anesthesia in children [9, 10]. However, electroencephalography-derived monitors are not always feasible in various clinical settings (e.g., magnetic resonance imaging) of pediatric procedural sedation. Therefore, establishing the relationship between the *C*_*e*_ of propofol and sedation scale will be helpful for targeted procedural sedation in pediatric patients.

We hypothesized that an adequate pharmacodynamic model between *C*_*e*_ of propofol and University of Michigan Sedation Scale (UMSS) can be made in children, and planned a prospective modeling study.

## Methods

### Patient recruitment and anesthetic methods

The study was approved by the Institutional Review Board of Seoul National University Hospital (Ref No; 1705–110-855). Written informed consent was obtained from one of the parents or legal guardians for minor patients, and patients were given a verbal explanation and had the opportunity to ask questions about the study methods and purposes. Informed consent was obtained from each patient. All procedures followed the principles of the Declaration of Helsinki and its subsequent revisions. This study was registered at http://www.clinicaltrials.gov (NCT03195686, Principal investigator; Hee-Soo Kim, Date of registration; 22/06/2017) prior to patient enrollment.

A total of 30 patients (aged 3 to 6 years) who underwent surgery under general anesthesia lasting more than 1 h were enrolled in this study. The exclusion criteria were obstructive sleep apnea, expected difficult airway management, or any conditions affected by propofol, such as mitochondrial diseases.

All patients fasted according to the guidelines of the American Society of Anesthesiologists. Baseline heart rate (HR) and noninvasive blood pressure (BP) were measured on admission. An intravenous route was established before transferring the patient to the operating theater, and patients received intravenous midazolam 0.1 mg/kg as premedication. Standard monitoring, including an electrocardiogram, HR, noninvasive BP at 1-min intervals, peripheral oxygen saturation (SpO_2_), and end-tidal carbon dioxide (E_T_CO_2_), was performed after arrival in the operating theater. A facial mask was applied, and oxygen (6 L/min) was administered. Lidocaine administration (0.5 mg/kg) was followed by propofol infusion with the effect site TCI mode (Kim and Choi’s model [16]) set at a *C*_*e*_ of 1 μg/mL. The propofol infusion line was connected just near the patient’s intravenous catheter site to minimize the dead space. After reaching the target, *C*_*e*_ was maintained for 2 min to ensure equilibration, and the target *C*_*e*_ was increased by 1 μg/mL for each step (up to *C*_*e*_ = 6 μg/mL). The Kim and Choi’s pediatric propofol model was recently developed and externally validated in previous studies [16, 25]. Administration of propofol was conducted using an infusion control software (ASAN pump program, http://fit4NM.org/d_asanpump, last accessed: 03 Nov, 2020) with a syringe pump (Pilot Anesthesia 2, Fresenius Kabi AG, Bad Homburg vdh, Germany). Detailed logs of infusion, including time and rate of infusion, were automatically recorded during the whole infusion period [26]. The criteria for determining the UMSS score are presented in Table 1.

**Table 1:** University of Michigan Sedation Scale

0	Awake and alert
1	Minimally sedated: tired/sleepy, appropriate response to verbal conversation and/or sound
2	Moderately sedated: somnolent/sleeping, easily aroused with light tactile stimulation or a simple verbal command
3	Deeply sedated: deep sleep, arousable only with significant physical stimulation
4	Unarousable

UMSS was assessed by an experienced pediatric anesthesiologist (Y.E.J.) who was blinded to the *C*_*e*_ of the propofol. Response to a verbal conversation (UMSS = 1, sleepy appropriate response to sound) was assessed by calling the patient’s name. Light tactile stimulation was applied (UMSS = 2, moderately sedated) by touching the patient’s eyebrows, and significant physical stimulation was applied (UMSS = 3, deeply sedated) by squeezing the trapezius. After the patient exhibited a UMSS = 4 (unarousable), rocuronium 0.6 mg/kg was administered to facilitate tracheal intubation, and remifentanil was started for general anesthesia. During anesthesia, the target *C*_*e*_ values for propofol and remifentanil were controlled by the attending anesthesiologists. Ventilation was adjusted to a tidal volume of 7 mL/kg, and the respiratory rate was adjusted to maintain E_T_CO_2_ of 35–40 mmHg; the inspired oxygen fraction was 0.4 in 2 L of fresh gas. Body temperature was continuously monitored and maintained above 35.5 °C with active warming.

On the closure of the surgical wound, propacetamol 15 mg/kg was administered for postoperative pain control. Remifentanil administration was halted 10 min before emergence and propofol administration was stopped by the attending anesthesiologist’s decision. In addition, HR, BP, SpO_2_, and E_T_CO_2_ were continuously monitored throughout the study period and anesthesia. When patients met the extubation criteria (UMSS = 0 or 1, train-of-four ratio > 0.9, and adequate tidal volume > 6 mL/kg), they were extubated and transferred to a post-anesthesia care unit (PACU). All adverse events (hypertension, BP increased more than 20% of baseline values; hypotension, BP decreased more than 20% of baseline values; tachycardia, HR increased more than 20% of baseline values; bradycardia, HR decreased more than 20% of baseline values; apnea, no spontaneous breathing for 15 s or desaturation; and < 95% of SpO_2_) were observed and recorded during the study period (from the induction of anesthesia to the discharge from PACU).

On completion of the study, the infusion history for propofol was obtained from the ASAN pump program. To eliminate the confounding effect of surgical pain and analgesic medications (remifentanil and propacetamol), only UMSS scores and the *C*_*e*_ values of propofol during induction of anesthesia were used for pharmacodynamic modeling of propofol sedation. Statistical analyses for descriptive statistics were performed using SPSS 23.0 for Windows (IBM SPSS Statistical Software, Chicago, IL, USA).

### Investigation of the relationship between C_e_ of propofol and sedation scale; pharmacodynamic modeling

To transfer ordinal UMSS scores into binary outcomes, we defined the UMSS score being equal to or greater than a given level *n* as a “response” and otherwise as a “non-response,” which were converted to 1 and 0, respectively. Logistic regression analysis was performed to examine the pharmacodynamic relationship between the *C*_*e*_ of propofol and UMSS scores. Referring to previous similar studies [27, 28], the probability of response to a given level of sedation score [*P* (UMSS) ≥ *n*] at a given *C*_*e*_ of propofol was analyzed using the following sigmoid *Emax* model:

$$P\ \left(\mathrm{UMSS}\ge n\right)=\frac{{C_e}^{\gamma }}{{C_{e50\mathrm{UMSS}\ge n}}^{\gamma }+{C_e}^{\gamma }},$$

where *C*_*e*50UMSS ≥ *n*_ is defined as a steady-state effect-site concentration of propofol with a 50% probability of a UMSS score equal to or greater than *n*, and γ is the Hill coefficient describing the slope steepness for the relationship of the *C*_*e*_ versus sedative response. The value of γ was assumed to be the same for all the sedation scores.

Based on the notion of “response” and “non-response” mentioned above, observation of a specific UMSS score *n* at a given *C*_*e*_ can be explained as the co-occurrence of a “response” for scores 0, 1, ..., *n* and a “non-response” for levels *n* + 1, …, 4. Therefore, the probability of observing the UMSS score of *n* at a given *C*_*e*_ can be calculated as the product of probabilities for “response” for levels 0, 1, …, *n* and “non-response” for levels *n* + 1, …, 4. Hence, *P* (UMSS = *n*) can be calculated as follows:

$$P\left(\mathrm{UMSS}=n\right)=\prod_{k=0}^nP\left(\mathrm{UMSS}\ge k\right)\times \prod_{k=n+1}^4\left(1-P\left(\mathrm{UMSS}\ge k\right)\right)$$

$$\left(0\le n\le 4\right).$$

By this estimation, we defined the predicted UMSS score for a given *C*_*e*_ as the score with the highest probability and compared predicted and observed scores.

### Model building and evaluation

We set our primary outcome as *C*_*e*50UMSS ≥ *n*_ and their relative standard errors obtained from the pharmacodynamic model. Secondary outcomes were distribution of UMSS scores against *C*_*e*_ of propofol, other derived parameters such as gamma, *P* (UMSS ≥ *n*), or *P* (UMSS = *n*) for each UMSS score from the model, demographic data, and incidence of adverse events such as desaturation, apnea, or hemodynamic instability. Among the data obtained during infusion of propofol, several points were selected, including the start of infusion and increment of UMSS score to 1, 2, 3, and 4. In addition, to reflect the change in *C*_*e*_ and obtain sufficient sample points, one additional point for each UMSS score was taken, which was set as the midpoint of the period in which the specific UMSS score was maintained, except for the score of 4. For UMSS score of 4, data at the time point when 20 s were elapsed after the score was changed were extracted.

The pharmacodynamic model was built using the Laplace method of NONMEM® 7.4.4 (ICON Development Solutions, Dublin, Ireland) via an interface program named Pirana® 2.9.9 (http://pirana-software.com, currently only provided by Certara, Princeton, NJ, USA). The NONMEM software calculated the likelihood (*L*) of the observed response on the UMSS score (*R*) as follows:

$$L=R\times P\ \left(\mathrm{UMSS}\ge n\right)+\left(1-R\right)\times \left[1-P\ \left(\mathrm{UMSS}\ge n\right)\right]$$

Inter-individual variability for each of *C*_*e*50UMSS ≥ *n*_ and gamma were estimated via a log-normal method or fixed to zero if necessary.

To evaluate model performance, we used the methods described by previous investigators to calculate the prediction probability (*P*_*k*_) of the predicted UMSS score given the observed UMSS score. With a pharmacokinetic tool program named fit4NM 4.6.0 (Eun-Kyung Lee and Gyu-Jeong Noh; http://www.fit4nm.org/download/246; last accessed: 03 Nov, 2020), *P*_*k*_ was calculated as follows [27–29]:

$${P}_k=\frac{\mathrm{Somer}{\mathrm{s}}^{\prime }\ d+1}{2},$$

where *P*_*k*_ = 1 indicates the complete agreement between the observed level of sedation and calculated index; *P*_*k*_ = 0.5 is the random relationship between the two; and *P*_*k*_ = 0 indicates complete disagreement.

In addition, we used a non-parametric bootstrap method to perform an internal validation of the model using the Perl-speaks-NONMEM (PsN) software ver. 4.9.0 (https://uupharmacometrics.github.io/PsN/). Bootstrapping was performed by resampling with the replacement of individuals to create a new dataset with an equal number of individuals as that in the original dataset (*n* = 30). Dataset formation and parameter estimation were repeated 1000 times. The 2.5–97.5% percentiles of the distribution of the parameter estimates across the nonparametric bootstrap replicates were used to build a 95% confidence interval and compared with the parameter estimates of the final model.

### Comparison with other pediatric models

As Kim and Choi’s model is not of widespread use nor commercially available, we compared the estimated *C*_*e*_ values from Kim and Choi’s model and from other commercially available models, which are the Kataria model [21] and the Schüttler’s model [30] used in the Paedfusor. As we have complete infusion history for each patient, we simulated the infusion and obtained estimated plasma concentration and *C*_*e*_ of propofol for every time point using PKPD tools for excel by C. Minto and T. Schnider (http://pkpdtools.com/excel/downloads/). We assumed actual use of the Kataria model and the Paedfusor model via commercially available Agilia® SP TIVA infusion pump (Software version 2.2, Fresenius Kabi, Fresenius Kabi AG, Bad Homburg vdh, Germany) and used pharmacokinetic-pharmacodynamic parameters based on literature [21, 31] and offered by the manufacturer. Afterward, we sorted estimated *C*_*e*_ values for time points included in the pharmacodynamic modeling and compared predictions from Kataria model or Paedfusor model with predictions from Kim and Choi’s model via the Bland-Altman plot using MedCalc® (ver. 20.008, MedCalc Software Ltd., Ostend, Belgium).

### Sample size calculation

Since this study was an exploratory study not intended to test a specific hypothesis, the calculation of the sample size was not necessary. We referred to a similar study on the pharmacodynamics of propofol study in terms of modified observer’s assessment of the alertness/sedation scale, in which data from 30 patients were used [32].

## Results

A total of 32 patients were recruited, and 30 completed the study. Incomplete data collection resulted in the loss of two patients from the study. Demographic data are presented in Table 2.

**Table 2:** Demographics and characteristics of the patients (*n* = 30)

Clinical variables	Values
Sex (M/F)	16/14
Age (years)	4.8 (0.95)
Height (cm)	109.6 (9.4)
Weight (kg)	19.2 (4.8)
Surgery	
Otolaryngeal surgery	19
Orthopedic surgery	3
Plastic surgery	2
Urologic surgery	3

Values are expressed as number or mean (standard deviation)

**Fig. 1:**
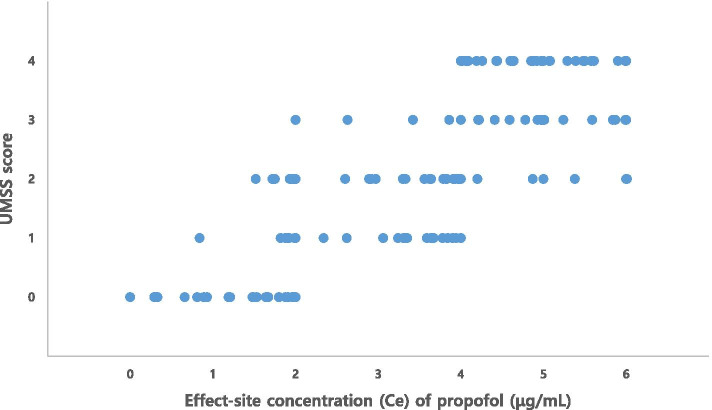
UMSS score vs *C*_*e*_ of propofol. This figure shows a scattered plot of observation of the University of Michigan Sedation Scale (UMSS) score versus effect-site concentration (*C*_*e*_) of propofol

The relationship between the *C*_*e*_ of propofol and corresponding UMSS scores is presented in Fig. 1. At a given UMSS score, the *C*_*e*_ of propofol varied between individuals.

All 237 pairs of *C*_*e*_ of propofol and UMSS score were used for the development of the model. The estimated population parameters of the *C*_*e50*_ of propofol at a given UMSS score and gamma are presented in Table 3. When the *C*_*e*_ of propofol increased, the depth of sedation was deeper and UMSS score was higher.

**Table 3:** Parameter estimates of the population pharmacodynamics model for *C*_*e*_ of propofol and UMSS score (n = 30)

Parameter	Estimate, (RSE, %), [95% CI] (μg/mL)	Median (2.5–97.5%) of bootstrap replicates (μg/mL)
*C*_*e50*UMSS ≥ *1*_	1.84 (8.3) [1.54–2.14]	1.85 (1.67–2.04)
*C*_*e50*UMSS ≥ *2*_	2.64 (8.6) [2.20–3.08]	2.66 (2.40–2.97)
*C*_*e50*UMSS ≥ *3*_	3.98 (4.1) [3.66–4.30]	4.00 (3.79–4.19)
*C*_*e50*UMSS ≥ *4*_	4.78 (2.7) [4.53–5.03]	4.81 (4.64–4.96)
γ	5.76 (15.6) [4.00–7.52]	5.78 (4.89–7.32)

Parameter estimates were obtained via the Laplace method using NONMEM® 7.4.4 (ICON Development Solutions, Dublin, Ireland). Inter-individual random variability was not assumed. Bootstrap analysis was repeated 1000 times. *C*_*e*_ Effect-site concentration, *UMSS* University of Michigan Sedation Scale, *C*_*e50*UMSS ≥ *n*_ Effect-site concentration of propofol with 50% probability of UMSS score equal to or greater than n, *RSE* Relative standard error

**Fig. 2:**
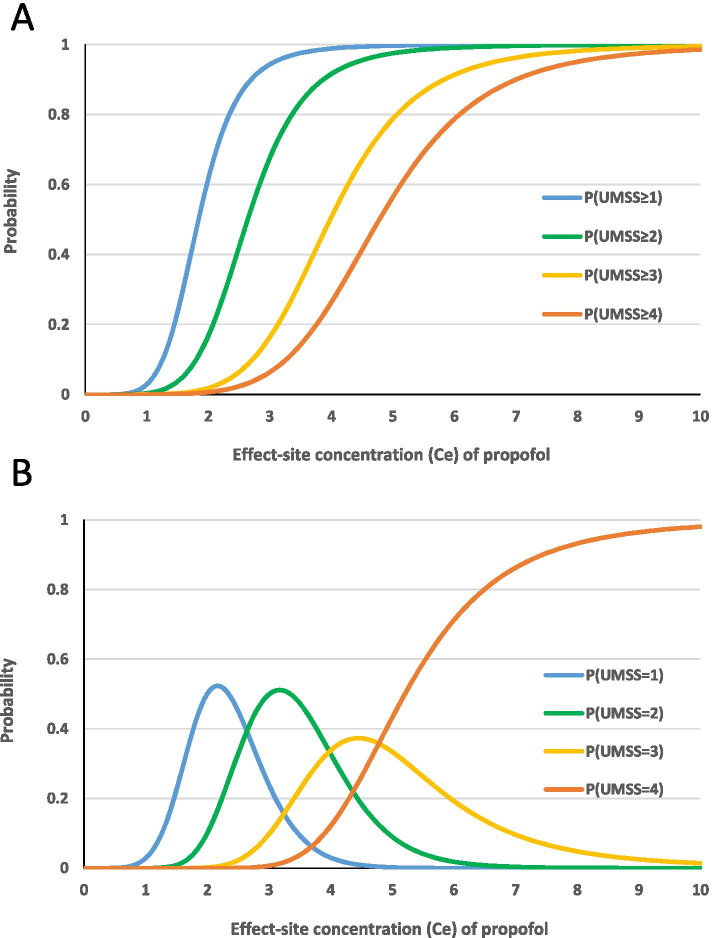
Estimation of probabilities of UMSS score. 2A depicts the estimation of the probability that the University of Michigan Sedation Scale (UMSS) score is *n* or more (*n* = 1, 2, 3, 4) according to the effect-site concentration (*C*_*e*_) of propofol. Probabilities were calculated for *C*_*e50*UMSS *≥ n*_ with a pharmacodynamic model using the Laplace method. 2B shows the probability for each specific UMSS score according to *C*_*e*_ of propofol. Detailed calculation methods are presented in the methods section. The probabilities for UMSS scores = 1, 2, and 3 show a single peak, and the probability of UMSS score = 4 shows a gradual increase as the *C*_*e*_ of propofol increases. *C*_*e50*UMSS *≥ n*_, the steady-state effect-site concentration of propofol with a 50% probability of UMSS score being equal to or greater than *n*

Figure 2 presents the probability of showing the degree of sedation corresponding to a given UMSS score or higher according to the *C*_*e*_ of propofol in children. In addition, the calculated probability for each specific UMSS score according to *C*_*e*_ of propofol is presented.

The predicted UMSS scores that had the highest probability according to changes in *C*_*e*_ are shown in Table 4.

**Table 4:** Predicted UMSS scores according to change in the *C*_*e*_ of propofol via Kim and Choi’s model

UMSS score	*C*_*e*_ (μg/mL)
0	*C*_*e*_ < 1.9
1	1.9 ≤ *C*_*e*_ < 2.7
2	2.7 ≤ *C*_*e*_ < 4.0
3	4.0 ≤ *C*_*e*_ < 4.8
4	*C*_*e*_ ≥ 4.8

Predicted UMSS scores were determined as the score with the highest probability at the given *Ce*

*UMSS* University of Michigan Sedation Scale, *C*_*e*_ Effect-site concentration

**Fig. 3:**
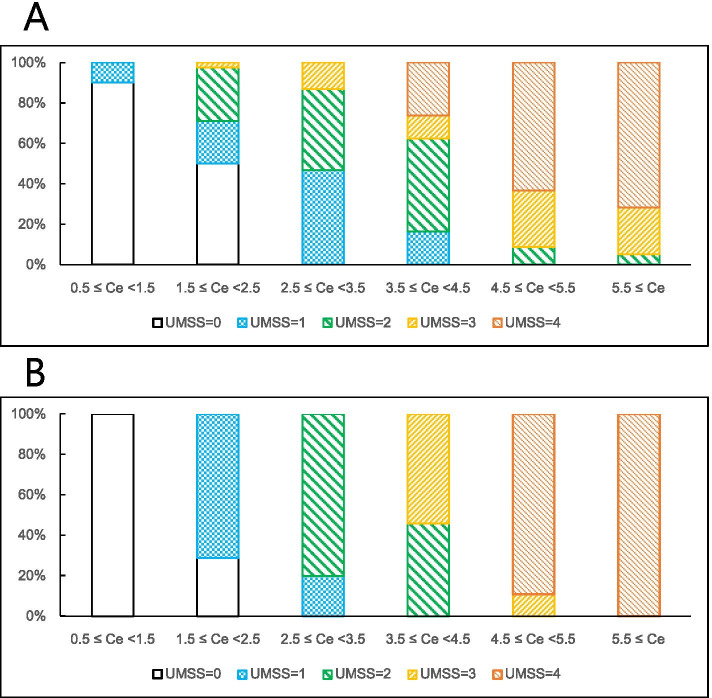
Observed vs predicted distribution of UMSS. The proportions of each observed and predicted University of Michigan Sedation Scale (UMSS) score according to the range of the effect-site concentration (*C*_*e*_) of propofol are shown. 3A is for observed and 3B is for predicted UMSS score. The predicted UMSS score was determined as the score with the highest probability for a given *C*_*e*_ of propofol. Each section of *C*_*e*_ is set such that the values rounded from the first decimal place are included in the same section

The calculated *P*_*k*_ (95% CI) was 0.770 (0.731–0.809). This value implies an excellent degree of agreement between the observed and predicted UMSS scores and, therefore, acceptable performance of the model. In addition, the observed and predicted distributions of the UMSS score according to the range of *C*_*e*_ are shown in Fig. 3.

**Fig. 4:**
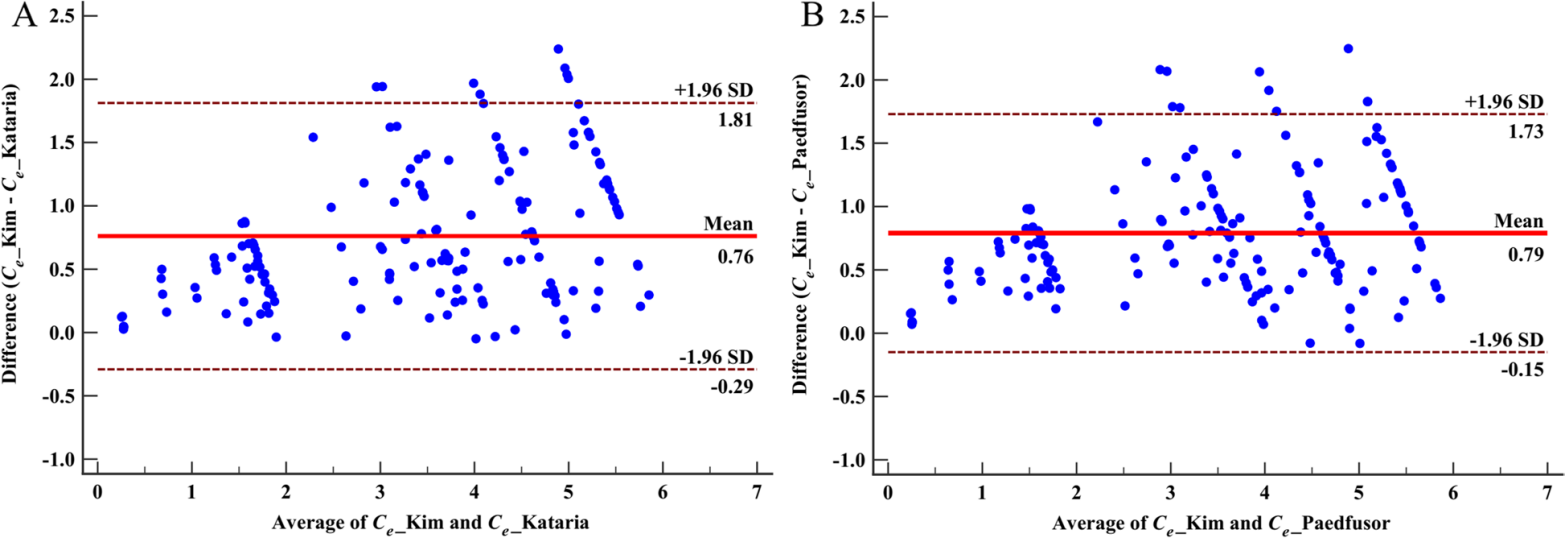
Comparison of predicted *C*_*e*_ of propofol between models. Bland-Altman plots showing the agreement between the estimation of *C*_*e*_ of propofol by the Kim and Choi’s model, and *C*_*e*_ of propofol by the Kataria model (4A) and by the Paedfusor model (4B). The differences were obtained by subtracting predictions by the Kataria model or the Paedfusor model from predictions by the Kim and Choi’s model. Lines for 95% limits of agreements were drawn

For comparison of commercially available models with the Kim and Choi’s model, data from 23 patients were used, since infusion logs for seven patients were flawed. Excluding points before the start of the infusion, total of 166 time-points were included. Figure 4 shows the Bland-Altman plot for differences in the estimated *C*_*e*_ of propofol among different models. The Kim and Choi’s model predicted the *C*_*e*_ of propofol to be higher than the Kataria model and the Paedfusor model, with biases (95% CI) of 24.2% (21.8–26.6%) and 27.9% (25.1–30.7%), respectively.

There were no complications such as respiratory depression after premedication with midazolam. During the induction of sedation, all patients were able to breathe spontaneously, and there were no desaturation events. At UMSS score = 4, five patients required jaw thrust to maintain airway patency with spontaneous breathing, but desaturation did not occur. No hypertension, hypotension, bradycardia, or tachycardia was observed in any patient. All patients were transferred to the PACU without any desaturation events or other respiratory complications after extubation and had a UMSS score = 1. In the PACU, no patients experienced apnea or desaturation or had unstable blood pressure or heart rate.

## Discussion

This study was the first to quantify the relationship between *C*_*e*_ of propofol and the sedation probability in children according to the UMSS score using the pharmacodynamic model. The findings of this study may provide important information based on which anesthesiologists can estimate the sedation probability in children as assessed by the UMSS at a given *C*_*e*_ of propofol.

Previous studies by McFarlan et al. [14] and Steur et al. [15] have suggested a manual propofol dosage scheme for total intravenous anesthesia in children aged 3–11 years and < 3 years, respectively. To produce a steady-state blood concentration of 3 μg/mL by manual infusion of propofol for children aged 3–11 years [14], a loading dose of 2.5 mg/kg was followed by continuous infusion of 15 mg/kg/hr for the first 15 min, 13 mg/kg/hr from 15 to 30 min, 11 mg/kg/hr from 30 to 60 min, 10 mg/kg/hr from 1 to 2 h, and 9 mg/kg/hr from 2 to 4 h. For children aged < 3 years, Steur and colleagues proposed different propofol dosage schemes for each four age group [15] (0–3 months, 3–6 months, 6–12 months, and 1–3 years), and they reduced the continuous infusion rate every 10 min to prevent delayed recovery.

This complexity of manual infusion is due to the pharmacological properties of propofol in pediatric patients [14, 15]. Younger children require higher induction and maintenance doses because of a greater volume of distribution and elevated systemic clearance [16]. Additionally, lighter children require a higher weight-based infusion rate of propofol to maintain a certain level of *C*_*e*_ [14–16]. The context-sensitive half time in children is significantly longer than that in adults, and it is increased by prolonged infusion [14]. Therefore, maintaining an appropriate level of sedation with the manual infusion of propofol is cumbersome and difficult in various clinical situations of pediatric sedation [10, 33].

TCI uses allometric scaling to describe age-related changes in the volume of distribution and metabolic clearance in pediatric patients of various ages [4, 16]. Changes in the pharmacological parameters are automatically calculated in pharmacokinetic models for more accurate drug delivery and reduced variability [11]. TCI is typically associated with less respiratory depression, less dose of propofol, and faster recovery [11]. In pediatric patients, TCI of propofol shows less variability in the BIS with less dose adjustment compared with that of manual infusion during general anesthesia [9, 10]. Therefore, TCI gives a more stable sedation than manual infusion does in pediatric patients.

A previous study investigated the population pharmacodynamics of midazolam and sedation score in adult patients after coronary artery bypass grafting [28]. The basic idea of the present study was adapted from this previous study. The clinical observational findings of our study were similar to those of this study. Although the drugs investigated in the studies (midazolam versus propofol) are different, the range of drug concentrations at a given sedation score varied, which is commonly observed during sedation (Fig. 1). Therefore, it would be reasonable to expect the probability of a certain sedation score at a given drug concentration.

In our final model, inter-individual variability for *C*_*e50*_ and gamma was fixed to zero. When we assumed inter-individual variability during the modeling, significant shrinkage, which was greater than 30%, occurred for all of the parameters except for the *C*_*e50*UMSS ≥ 2_, while not substantially reducing the objective function value. As a high level of shrinkage is indicative of a high level of estimation error [34], we decided not to assume inter-individual variability. Therefore, we used a naïve pooled data approach that assumes inter-individual variability as zero, and accordingly, within subject correlation was not assumed. A similar method has been used in previous studies [27, 32]. The bootstrap results showed fair agreement with the original estimates from the final model.

We did not present classical tools for model performance such as the goodness-of-fit plot or visual predictive check in this study. Such methods were difficult to apply in this model because the observed UMSS scores were ordinal variables rather than continuous variables and were expressed as integers, whereas the predicted values were completely different probability values. Although we presented the distribution of predicted UMSS scores in Fig. 3, it was only intended to provide an interpretation to aid the application of our model in clinical settings.

In pediatric patients, Munoz et al. investigated the *C*_*e*_ of propofol required to produce hypnosis in children aged 3–11 years using BIS monitoring [35]. In that study, the mean ${EC}_{e_{50}}$ for hypnosis assessed at BIS value = 50 was 3.65 μg/mL. Additionally, a retrograde study of propofol sedation with the Paedfuor plasma TCI model in children aged < 7 years reported that the target *C*_*e*_ of propofol for long-duration of immobilization and spontaneous ventilation was 4.3 μg/mL during proton radiation therapy [12]. These values matched the 3.98 [3.66–4.30] μg/mL of *C*_*e50*_, which corresponded to a UMSS = 3, in our study. From the results of these two studies, we inferred that a UMSS = 3 may correspond to a BIS value of 50 and can produce deep sedation for long-duration radiologic procedures.

Based on the present study (Table 2), we suggest an initial target *C*_*e*_ of 1.5–2.0 μg/mL for minimal sedation (UMSS = 1), 2.0–3.0 μg/mL for moderate sedation with light tactile stimuli (UMSS = 2), and 3.5–4.0 μg/mL for deep sedation with significant physical stimuli or require immobilization (UMSS = 3). The *C*_*e*_ of propofol should be adjusted for specific procedure-related stimulation, such as noise, tactile stimuli, and pain because the depth of sedation and degree of respiratory depression can change accordingly. Because the depth of sedation by the UMSS score changes rapidly in the *C*_*e*_ range of 2.0–4.0 μg/mL, and the change cannot be predicted 100% accurately, we suggest that the concentration should be more precisely controlled with a smaller incremental dose and close observation of the patient. Consequently, monitoring the depth of sedation using electroencephalography-based devices would be helpful for propofol-induced sedation in pediatric patients [9].

On comparison of the Kim and Choi’s model with commercially available Kataria model and Paedfusor model, the Kim and Choi’s model predicts the *C*_*e*_ to be about 24 to 28% higher than other models. Considering that bias within 30% is usually regarded acceptable when evaluating performance of pharmacokinetic-pharmacodynamic models, we can say that our model for UMSS score can be applied to other popular models such as the Kataria model and the Paedfusor model. Still, we want to recommend using slightly lower target *C*_*e*_ values when using models on the market, along with continuous feedback from patients’ observed state of sedation.

This study has several limitations. First, the relationship between the *C*_*e*_ of propofol and UMSS score was evaluated during general anesthesia, not during procedural sedation. To eliminate various confounding effects, this model used data only during anesthesia induction. As TCI ensures a certain *C*_*e*_ of propofol, the UMSS score did not change during a constant target *C*_*e*_ of propofol. Therefore, although this study lacks data from during the maintenance of sedation, the pharmacodynamic model can provide meaningful information for pediatric sedation using propofol TCI. Second, the depth of anesthesia was not measured with electroencephalography-based monitors in this study. Additionally, the depth of sedation after reaching the UMSS = 4 could not be assessed. To minimize the bias in estimating the *C*_*e50*_ of propofol at UMSS = 4, we used the *C*_*e*_ of propofol when the patient reached the UMSS = 4 and 20 s after the subject reached the UMSS = 4. Third, the plasma concentration of propofol was not measured in this study. As minimal premedication was given to the patients of this study, serial blood sampling was not possible. Kim and Choi’s pediatric propofol model, which was used in this study, was externally validated and showed good performance in achieving the target propofol plasma concentration in children aged < 12 years [16, 25]. Also, as mentioned above, prediction of *C*_*e*_ values with the Kim and Choi’s model is acceptable when regarding predictions from previous pediatric models as gold standard. Finally, premedication with intravenous midazolam could affect the sedation score given the *C*_*e*_ of propofol. However, 0.1 mg/kg of midazolam was not sufficient to cause a significant sedative effect, and as premedication with midazolam has been widely performed in general anesthesia and procedural sedation in children [36], it would not be different from clinical practice.

## Conclusions

In conclusion, the relationship between *C*_*e*_ of propofol and UMSS score with probability by the population pharmacodynamic approach in children was established. This finding may be helpful in predicting the depth of sedation when using TCI of propofol in various pediatric procedural sedation settings.

## Data Availability

The datasets generated during and analysed during the current study are not publicly available since we did not store the data at a public web-based archive, but are available from the corresponding author on reasonable request.

## Abbreviations

BP: Blood pressure
BIS: Bispectral index
*C*_*e*_: Effect site concentration
E_T_CO_2_: End-tidal carbon dioxide
HR: Heart rate
PACU: Postanesthetic care unit
*P*_*k*_: Prediction probability
SpO_2_: Peripheral oxygen saturation
TCI: Target controlled infusion
UMSS: University of Michigan Sedation Scale
